# Association Between Coronary Artery Calcium Score and Right Ventricular Dysfunction: Insights from Combined Echocardiographic and CT Assessment

**DOI:** 10.3390/jcm15166318

**Published:** 2026-08-15

**Authors:** Davut Unsal Capkan, Mehmet Kaplan

**Affiliations:** 1 Department of Radiology, Medical Point Hospital, Gaziantep 27060, Turkey; 2 Department of Cardiology, Medical Point Hospital, Gaziantep 27060, Turkey; drkaplanmehmet@gmail.com

**Keywords:** coronary artery calcium score, right ventricular dysfunction, TAPSE, right ventricular fractional area change, coronary artery disease, echocardiography, cardiovascular risk stratification

## Abstract

**Background:** Coronary artery calcium score (CACS) is a well-established marker of coronary atherosclerotic burden and cardiovascular risk. While its association with left ventricular dysfunction has been extensively investigated, the relationship between CACS and right ventricular (RV) function remains insufficiently explored. This study aimed to evaluate the association between CACS and echocardiographic RV function parameters and to explore the discriminatory ability of CACS for identifying patients meeting predefined echocardiographic thresholds of RV dysfunction in patients with suspected stable coronary artery disease (CAD). **Methods:** This retrospective observational study included 96 patients who underwent coronary computed tomography angiography and transthoracic echocardiography within a 3-month interval. CACS was calculated using the Agatston method. RV function was assessed using tricuspid annular plane systolic excursion (TAPSE), right ventricular fractional area change (RV-FAC), and tissue Doppler-derived systolic velocity (S′). Correlation analyses, subgroup comparisons, multivariable linear regression, receiver operating characteristic (ROC) analyses, decision curve analysis (DCA), and calibration analyses were performed. **Results:** CACS demonstrated significant inverse correlations with TAPSE (r = −0.42, *p* < 0.001), RV-FAC (r = −0.36, *p* = 0.002), and S′ (r = −0.31, *p* = 0.006). Patients with higher CACS values exhibited progressively impaired RV systolic function. In multivariable regression analyses, higher CACS remained associated with lower TAPSE, RV-FAC, and S′ after adjustment for age, hypertension, and diabetes mellitus. Exploratory ROC analyses demonstrated moderate discriminatory performance, with AUC values ranging from 0.70 to 0.76. DCA suggested a potential net benefit across a range of threshold probabilities, while bootstrap calibration analysis demonstrated acceptable agreement between predicted and observed outcomes. **Conclusions:** In this retrospective single-center cohort, higher CACS values were associated with lower conventional echocardiographic measures of right ventricular systolic function. However, given the selected study population, relatively small sample size, limited clinical and instrumental characterization, potential residual confounding, and absence of external validation, these findings should be considered exploratory. The present data do not establish CACS as a clinical predictor of right ventricular dysfunction or support its use for clinical decision-making.

## 1. Introduction

Coronary artery disease (CAD) remains a leading cause of cardiovascular morbidity and mortality worldwide, making accurate detection of subclinical atherosclerosis and appropriate risk stratification essential for preventive management [1,2,3,4]. Coronary computed tomography provides a non-invasive assessment of coronary atherosclerotic burden, and the coronary artery calcium score (CACS) is widely used to estimate the risk of cardiovascular mortality and major adverse cardiovascular events [5,6]. In addition to its established prognostic role, increasing evidence suggests that coronary calcification may be associated with structural and functional myocardial abnormalities [7,8].

CACS reflects the cumulative effect of coronary atherosclerosis and is increasingly incorporated into clinical decision-making algorithms, especially for risk stratification in asymptomatic and stable patients. Higher CACS values have been consistently associated with an increased risk of myocardial infarction, cardiovascular mortality, and overall adverse cardiac outcomes [9,10,11].

Although cardiovascular imaging studies have traditionally focused on left ventricular function, right ventricular (RV) function is increasingly recognized as an important determinant of prognosis in several cardiovascular disorders [12,13,14]. RV dysfunction may occur in ischemic heart disease and can provide incremental prognostic information beyond conventional left-sided measurements. Transthoracic echocardiography is the most commonly used technique for RV assessment because of its accessibility and clinical applicability. Tricuspid annular plane systolic excursion (TAPSE), right ventricular fractional area change (RV-FAC), and tissue Doppler-derived systolic velocity (S′) are routinely used indices of RV systolic performance [15,16,17].

Previous studies have mainly investigated the relationship between CACS and left ventricular remodeling or diastolic dysfunction [18,19,20]. However, the potential impact of coronary calcification on RV mechanics remains largely unexplored. This gap in the literature is noteworthy, given that subclinical RV dysfunction may precede overt cardiac disease and could serve as an early marker of adverse cardiovascular remodeling.

Emerging evidence suggests that systemic atherosclerosis and coronary calcification may influence myocardial structure and function through mechanisms such as microvascular dysfunction, increased ventricular stiffness, and myocardial ischemia. These pathophysiological processes may not be limited to the left ventricle but could also affect the right ventricle, particularly through interventricular dependence and shared myocardial perfusion pathways [21]. Furthermore, RV dysfunction has been shown to be an independent predictor of morbidity and mortality across a wide spectrum of cardiovascular diseases, underscoring the need for its comprehensive evaluation [22].

Investigating the association between CACS and RV function may provide new insights into the impact of coronary atherosclerosis on cardiac performance. Furthermore, integrating radiological and echocardiographic findings may improve cardiovascular risk assessment.

Therefore, this study aimed to describe the distribution of coronary calcium and to evaluate the preliminary associations of total and territory-specific CACS with conventional echocardiographic indices of RV systolic function in patients undergoing evaluation for suspected stable CAD. Secondary exploratory analyses examined the within-sample discriminatory ability of CACS across predefined echocardiographic thresholds of RV dysfunction.

## 2. Materials and Methods

### 2.1. Study Design and Population

The study was designed primarily as an exploratory observational analysis of associations and not as the development or validation of a clinical prediction model. The study protocol was reviewed and approved by the Gaziantep City Hospital Non-Interventional Clinical Research Ethics Committee (Approval No: 375/2025, Date: 17 December 2025). The study was conducted in accordance with the principles outlined in the Declaration of Helsinki. Written informed consent was obtained from all participants prior to their inclusion in the study.

This retrospective observational study was conducted to evaluate the relationship between coronary artery calcium score (CACS) and right ventricular (RV) function in patients with suspected stable coronary artery disease (CAD). The study population consisted of adult patients who underwent coronary computed tomography angiography (CCTA) and transthoracic echocardiography (TTE) within a defined time interval.

Patients were identified through the hospital electronic medical record system. Individuals aged ≥18 years who were evaluated for suspected stable CAD and had both CACS measurements and echocardiographic RV parameters available were considered eligible. To ensure temporal consistency, only patients who underwent TTE within 3 months of CCTA were included. Both coronary computed tomography angiography (CCTA) and transthoracic echocardiography (TTE) had been performed as part of routine clinical care before study initiation. No imaging examinations were performed specifically for this study. The study consisted exclusively of a retrospective analysis of existing clinical and imaging data.

### 2.2. Inclusion and Exclusion Criteria

Patients were eligible for inclusion if they were aged ≥18 years, were evaluated for suspected stable coronary artery disease (CAD), had undergone coronary computed tomography angiography with an available coronary artery calcium score (CACS), and had transthoracic echocardiography performed within three months of CT imaging. Additionally, only patients with complete echocardiographic right ventricular (RV) function parameters, including tricuspid annular plane systolic excursion (TAPSE), right ventricular fractional area change (RV-FAC), and systolic myocardial velocity (S′), as well as fully accessible clinical and demographic data, were included in the study. Patients were excluded if they presented with acute coronary syndrome (ST-segment elevation myocardial infarction [STEMI], non-ST-segment elevation myocardial infarction [NSTEMI], or unstable angina), had a history of prior coronary revascularization (coronary artery bypass grafting or percutaneous coronary intervention), or had conditions known to significantly affect RV function, such as congenital heart disease, previously documented advanced pulmonary hypertension based on available clinical records and echocardiographic reports, significant tricuspid valve disease. Furthermore, patients with incomplete or poor-quality imaging data, pregnant or lactating women, and individuals who declined diagnostic procedures or did not provide informed consent were also excluded. All eligible patients were analyzed as a single study cohort. Correlation and multivariable regression analyses evaluating the association between CACS and echocardiographic right ventricular function parameters constituted the primary analyses. Stratification according to CACS categories (0–99, 100–399, and ≥400) was performed only as a secondary descriptive analysis to facilitate clinical interpretation of the findings.

### 2.3. Data Collection and Analysis

Clinical, demographic, radiological, and echocardiographic data were retrospectively collected from the institutional electronic medical record system and imaging archives. Demographic variables included age and sex, while clinical data comprised comorbidities such as hypertension, diabetes mellitus, dyslipidemia, chronic obstructive pulmonary disease, and chronic kidney disease, as well as the use of cardiovascular medications (e.g., beta-blockers, angiotensin-converting enzyme inhibitors). Radiological data were obtained from coronary computed tomography angiography, including total coronary artery calcium score (CACS) calculated using the Agatston method and segmental calcium distribution across major coronary arteries (left main, left anterior descending, circumflex, and right coronary artery). Echocardiographic data were extracted from transthoracic echocardiography reports and included right ventricular (RV) functional parameters such as tricuspid annular plane systolic excursion (TAPSE), right ventricular fractional area change (RV-FAC), and systolic myocardial velocity (S′), along with additional structural measurements including RV basal diameter and right atrial dimensions. To ensure temporal consistency, only patients with echocardiographic evaluation performed within three months of coronary CT imaging were included. All available data were reviewed for completeness and accuracy prior to statistical analysis.

### 2.4. Radiological Parameters (CCTA Data)

Coronary computed tomography angiography (CCTA) was performed using a 640-slice multidetector CT scanner (Aquilion ONE, Canon Medical Systems, Otawara, Japan) according to standard imaging protocols. Coronary artery calcium score (CACS) was quantified using the Agatston scoring method on a dedicated workstation (Vitrea Advanced Visualization Workstation, Vital Images Inc., Minnetonka, MN, USA), providing a standardized measure of coronary calcified plaque burden [23]. Total CACS was recorded for each patient, and segmental calcium scores were assessed separately for the left main (LM), left anterior descending (LAD), left circumflex (LCx), and right coronary artery (RCA). A coronary territory was considered involved when its corresponding segmental Agatston score was >0, and the number of territories with detectable calcification was recorded for each patient. For secondary exploratory analyses, patients were stratified according to total CACS as low (0–99), moderate (100–399), or high (≥400). Territory-specific measures were used to characterize the anatomical distribution of coronary calcification and were not interpreted as surrogate measures of coronary stenosis severity or lesion-specific ischemia. All imaging data were reviewed and analyzed by a radiologist with 22 years of experience in cardiac imaging, who was blinded to the echocardiographic findings to minimize observer bias.

### 2.5. Echocardiographic Assessment

Transthoracic echocardiography (TTE) was performed using a commercially available ultrasound system (Vivid E9, GE Healthcare, Chicago, IL, USA) in accordance with current guideline recommendations for right heart assessment [24]. All examinations were conducted with patients in the left lateral decubitus position, and standard parasternal and apical views were obtained. Right ventricular (RV) function was primarily evaluated from the apical four-chamber view focused on the RV.

Tricuspid annular plane systolic excursion (TAPSE) was measured using M-mode by placing the cursor through the lateral tricuspid annulus and recording the longitudinal displacement during systole; a value < 17 mm was considered indicative of RV systolic dysfunction. Right ventricular fractional area change (RV-FAC) was calculated as [(RV end-diastolic area − RV end-systolic area)/RV end-diastolic area] × 100, with values < 35% defined as impaired RV systolic function. Systolic myocardial velocity (S′, cm/s) was obtained using tissue Doppler imaging (TDI) at the lateral tricuspid annulus, reflecting longitudinal RV systolic performance.

In addition to functional parameters, structural measurements including RV basal diameter and right atrial dimensions were recorded. An RV basal diameter > 42 mm was considered indicative of RV dilation. Echocardiographic examinations had been performed during routine clinical care by a cardiologist with 10 years of experience in echocardiography. For the purpose of this study, the stored echocardiographic images and reports were retrospectively reviewed, and the quantitative measurements were confirmed by the same cardiologist, who was blinded to the CCTA findings.

To assess measurement reproducibility, 30 patients were randomly selected for repeat analysis. For the interobserver assessment, CACS, TAPSE, RV-FAC, and S′ were independently remeasured by a second observer from the same archived CT and echocardiographic image datasets using the original measurement protocols, while blinded to the initial results. For the intraobserver assessment, the respective primary observers repeated the measurements from the same archived images after a 4-week interval without access to their initial results. No new CT or echocardiographic examinations were performed for the reproducibility analysis. Interobserver and intraobserver agreement were assessed using intraclass correlation coefficients (ICC) with a two-way random-effects model and absolute agreement definition. ICC values < 0.50 were considered poor, 0.50–0.75 moderate, 0.75–0.90 good, and >0.90 excellent reproducibility.

### 2.6. Statistical Analysis

All statistical analyses were performed using IBM SPSS Statistics version 27.0 (IBM Corp., Armonk, NY, USA) and R software (version 4.3.0; R Foundation for Statistical Computing, Vienna, Austria). Continuous variables were expressed as mean ± standard deviation or median (minimum–maximum), depending on data distribution, while categorical variables were presented as frequencies and percentages. The normality of continuous variables was assessed using the Shapiro–Wilk test. The relationship between coronary artery calcium score (CACS) and right ventricular (RV) function parameters, including tricuspid annular plane systolic excursion (TAPSE), right ventricular fractional area change (RV-FAC), and systolic myocardial velocity (S′), was evaluated using Pearson correlation analysis for normally distributed variables and Spearman rank correlation for non-normally distributed variables. Comparisons of RV function parameters across CACS categories (0–99, 100–399, and ≥400) were performed using one-way analysis of variance (ANOVA) for normally distributed data or the Kruskal–Wallis test for non-parametric data. To identify factors independently associated with RV function parameters, multivariable linear regression analyses were performed including CACS, age, hypertension, diabetes mellitus, and other clinically relevant covariates as independent variables. Given the relatively small sample size, the multivariable models were specified parsimoniously a priori to minimize the risk of overfitting. Age, hypertension, and diabetes mellitus were included as potential confounders because they may be associated with both coronary calcification and right ventricular function. These models therefore estimated associations adjusted only for the prespecified available covariates and were not intended to establish CACS as an independent predictor of RV dysfunction. Although age, hypertension, and diabetes mellitus influence the development and progression of coronary calcification, they are not mathematical components of the Agatston score. Left ventricular systolic and diastolic function parameters and several other major determinants of RV function were not consistently recorded in the retrospective dataset and therefore could not be included in the models; consequently, residual confounding cannot be excluded. Candidate variables were selected a priori based on established clinical relevance and data availability. Internal validation of the prediction model was performed using bootstrap resampling with 1000 repetitions. Bootstrap calibration was used to assess model optimism and to estimate the calibration intercept, calibration slope, and Brier score. Interobserver and intraobserver reproducibility were evaluated using intraclass correlation coefficients (ICC) with corresponding 95% confidence intervals. Statistical significance was defined as a two-tailed *p*-value < 0.05. Sample size estimation was performed using G*Power software (version 3.1) based on the primary objective of evaluating the correlation between CACS and right ventricular function parameters. Assuming a moderate effect size (r = 0.50), a two-sided α level of 0.05, and a statistical power of 80%, the minimum required sample size was estimated to be 29 patients. The present study exceeded this requirement by including 96 patients. However, the additional multivariable regression, ROC, calibration, decision curve, subgroup, and reproducibility analyses should be considered exploratory because the study was not specifically powered for these secondary analyses.

**Figure 1:**
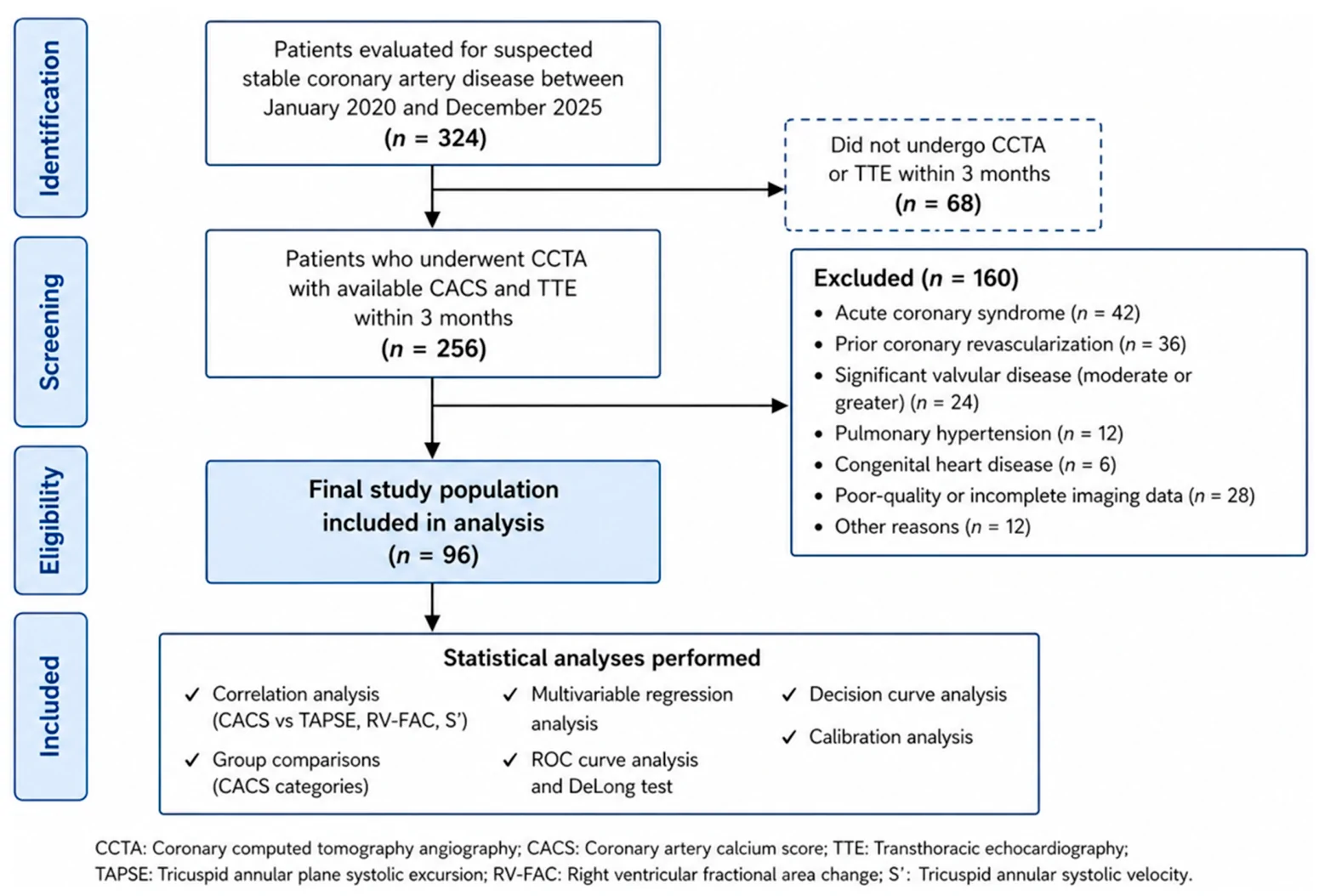
Flowchart of the study. Other reasons for exclusion (*n* = 12) comprised pregnancy or lactation, incomplete clinical or demographic records despite available imaging, and lack of informed consent.

The patient selection process and study flow are presented in Figure 1.

## 3. Results

The study population consisted of 96 patients with a mean age of 58.4 ± 10.7 years. Of these, 62 (64.6%) were male and 34 (35.4%) were female. The mean body mass index was 27.8 ± 4.2 kg/m^2^. Hypertension was present in 54 patients (56.3%), diabetes mellitus in 29 (30.2%), dyslipidemia in 47 (49.0%), chronic kidney disease in 12 (12.5%), and chronic obstructive pulmonary disease in 10 (10.4%). Regarding smoking status, 28 patients (29.2%) were current smokers, 22 (22.9%) were former smokers, and 46 (47.9%) were never smokers. In terms of medication use, beta-blockers were used by 51 patients (53.1%), ACE inhibitors or angiotensin receptor blockers by 49 (51.0%), statins by 45 (46.9%), antiplatelet agents by 52 (54.2%), and calcium channel blockers by 21 (21.9%). Smoking status was subclassified as current smoker, former smoker, and never smoker. Descriptively, current and former smoking were more frequent among men, whereas never smoking was more frequent among women. No statistically significant sex-based differences were observed in age, body mass index, comorbidities, or cardiovascular medication use (all *p* > 0.05) (Table 1).

The median interval between coronary computed tomography angiography (CCTA) and transthoracic echocardiography (TTE) was 27 days (interquartile range [IQR], 14–46 days). Exploratory analyses demonstrated no significant correlations between the imaging interval and TAPSE (r = −0.06, *p* = 0.54), RV-FAC (r = −0.05, *p* = 0.63), or S′ (r = −0.04, *p* = 0.71).

**Figure 2:**
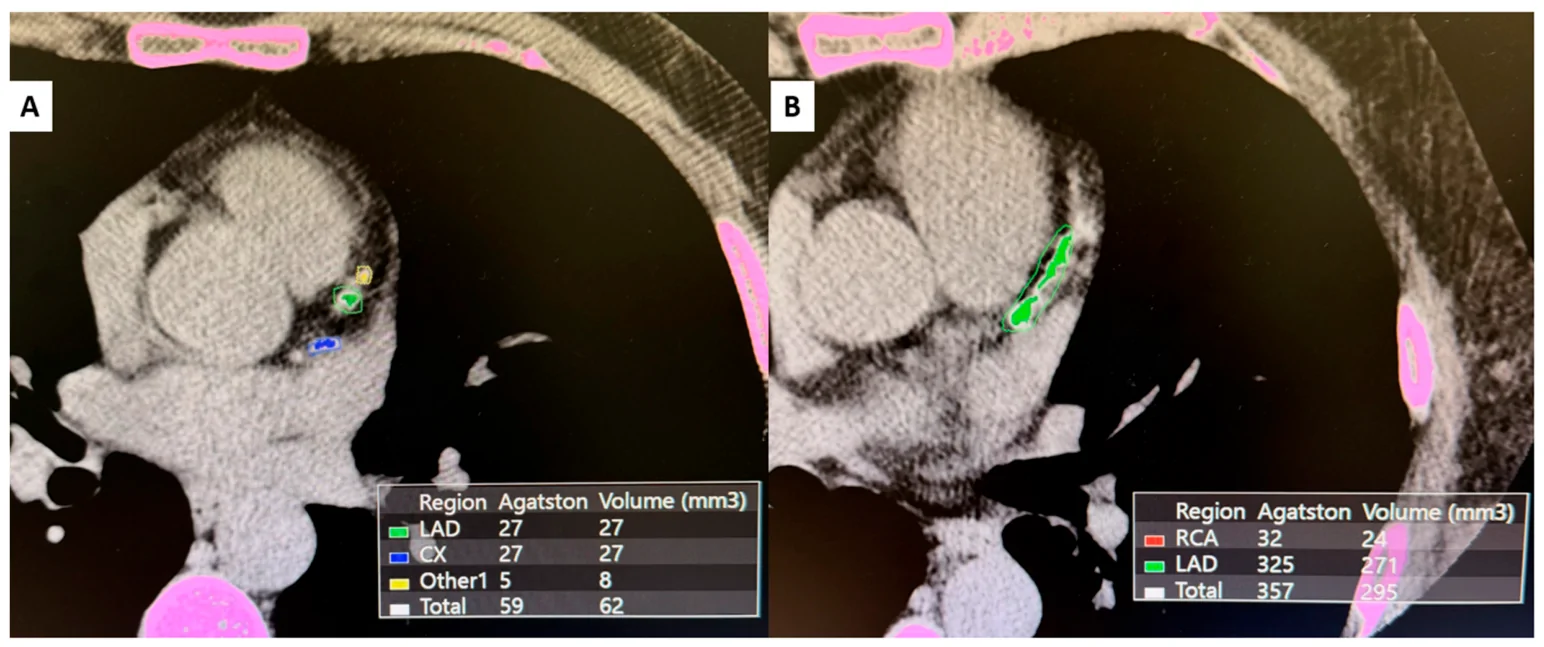
Representative coronary computed tomography images demonstrating coronary artery calcium scoring. (**A**) Example of mild coronary calcification with low Agatston score. (**B**) Example of severe coronary calcification with high Agatston score. Color-coded regions indicate calcified plaques in different coronary arteries.

The median total CACS was 42 (IQR, 5–185; range, 0–850). Overall, 64 patients (66.7%) were classified in the low CACS group, including 18 (18.8%) with no detectable coronary calcium, 22 (22.9%) with minimal calcification, and 24 (25.0%) with mild calcification. Moderate and high CACS values were observed in 20 (20.8%) and 12 (12.5%) patients, respectively. The LAD was the most frequently involved coronary territory (72.9%), followed by the RCA (44.8%), LCx (34.4%), and LM (15.6%). Among the 78 patients with detectable coronary calcium, 28 had one involved territory, 25 had two, 17 had three, and 8 had involvement of all four territories. The highest segmental calcium burden was observed in the LAD, with a median segmental CACS of 60 (IQR, 18–185) among patients with LAD involvement (Table 2, Figure 2).

Echocardiographic assessment demonstrated that the mean TAPSE value was 18.4 ± 2.1 mm, indicating generally preserved right ventricular systolic function across the study population. The mean RV fractional area change (RV-FAC) was 38.7 ± 6.2%, while the systolic myocardial velocity (S′) measured by tissue Doppler imaging was 16.0 ± 6.0 cm/s. The mean right ventricular basal diameter was 34.9 ± 3.8 mm. Right atrial dimensions were within expected ranges, with a mean major axis of 44.2 ± 3.1 mm, minor axis of 32.8 ± 3.5 mm, and an average area of 20.4 ± 2.3 cm^2^ (Table 3).

Comparison of RV function parameters across CACS categories revealed significant differences among groups. Patients with higher CACS values demonstrated progressively lower TAPSE and RV-FAC values, indicating impaired RV systolic function. Specifically, TAPSE decreased from 19.1 ± 1.8 mm in the low CACS group to 16.5 ± 2.0 mm in the high CACS group (*p* = 0.001). RV-FAC values were significantly reduced in patients with higher CACS (*p* = 0.003). Tissue Doppler-derived S′ values also showed a decreasing trend across increasing CACS categories (*p* = 0.012). In contrast, structural parameters such as RV basal diameter and right atrial area increased significantly with higher CACS levels (*p* = 0.021 and *p* = 0.018, respectively) (Table 4).

**Figure 3:**
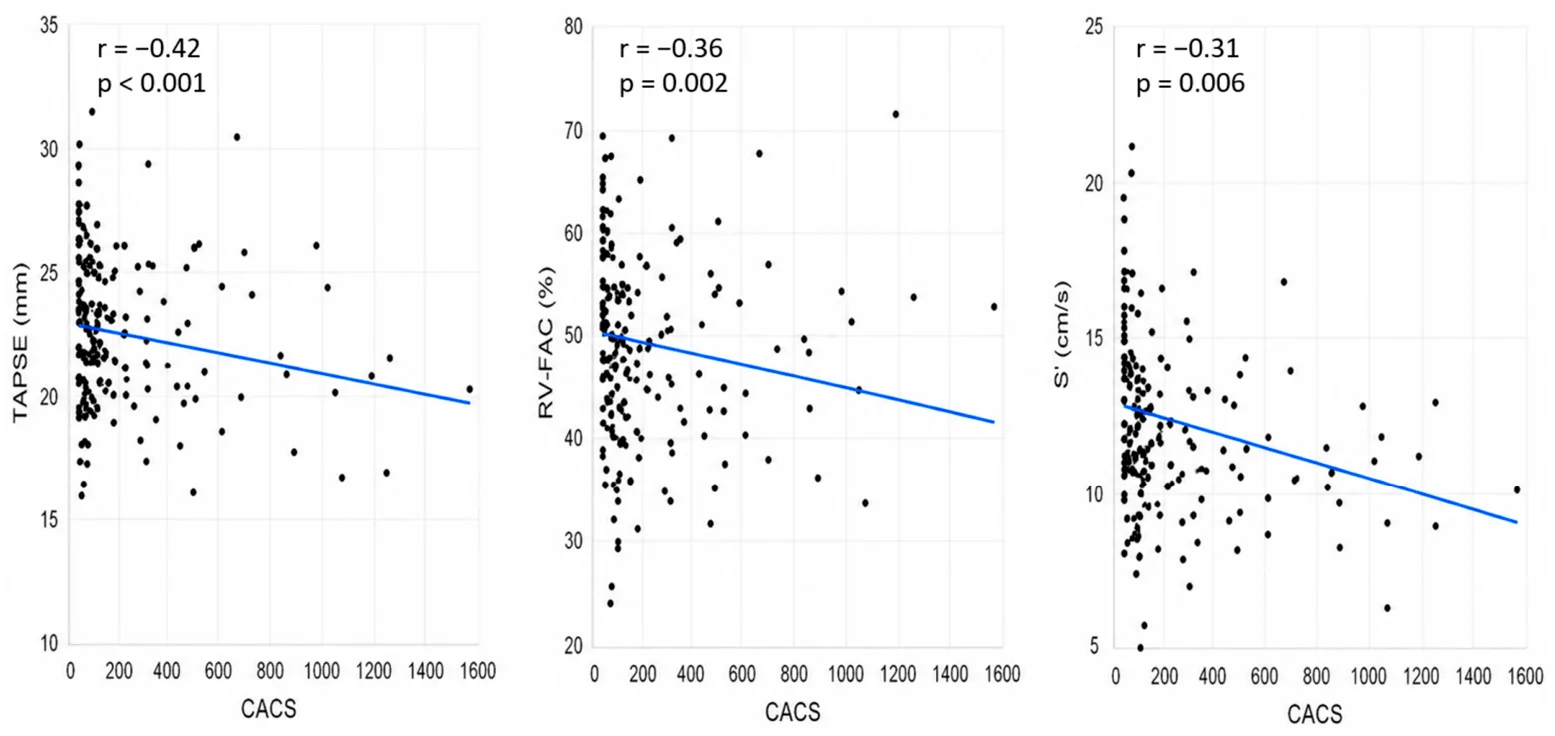
Correlation between coronary artery calcium score (CACS) and right ventricular function parameters (TAPSE, RV-FAC, and S′).

Correlation analysis demonstrated a significant inverse relationship between CACS and RV function parameters. Specifically, CACS was negatively correlated with TAPSE (r = −0.42, *p* < 0.001), indicating reduced longitudinal RV systolic function with increasing coronary calcification. RV-FAC showed a moderate negative correlation with CACS (r = −0.36, *p* = 0.002), while tissue Doppler-derived S′ values also decreased significantly as CACS increased (r = −0.31, *p* = 0.006) (Figure 3).

After adjustment for age, hypertension, and diabetes mellitus, higher CACS remained associated with lower TAPSE (β = −0.38, *p* < 0.001), RV-FAC (β = −0.33, *p* = 0.002), and S′ (β = −0.29, *p* = 0.006). The models had adjusted R^2^ values ranging from 0.24 to 0.34, and VIF values were below 2 for all included variables. However, because left ventricular function, pulmonary artery pressure, coronary stenosis severity, and other potential determinants of RV function were not systematically available, these estimates represent partially adjusted associations and should not be interpreted as evidence that CACS is an independent predictor of RV dysfunction (Table 5).

**Figure 4:**
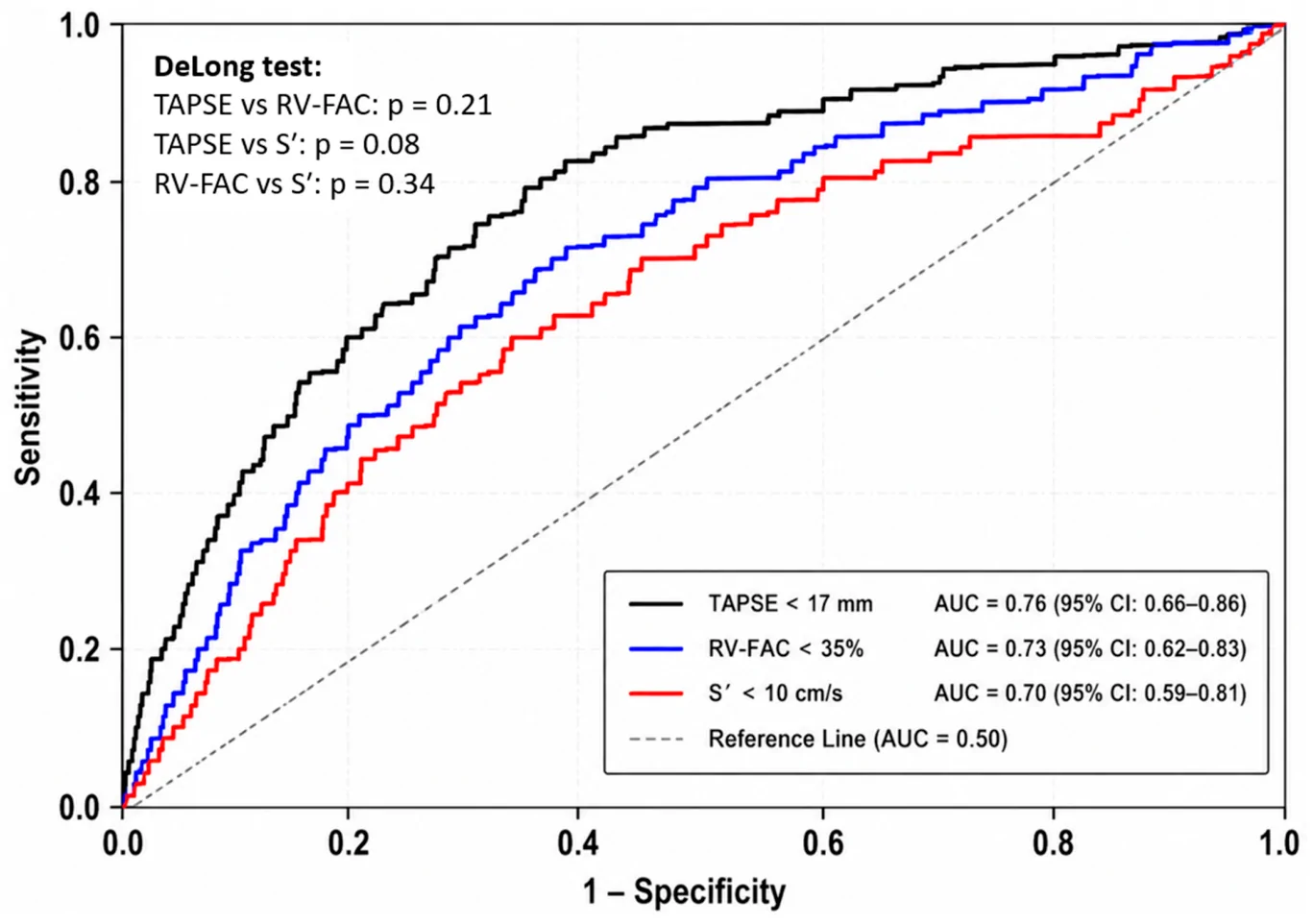
ROC curves evaluating the discriminatory performance of CACS for predefined echocardiographic thresholds of RV dysfunction.

Exploratory ROC analyses evaluated the ability of CACS to discriminate between patients with and without RV dysfunction according to the predefined echocardiographic thresholds. The AUC for predicting TAPSE < 17 mm was 0.76 (95% CI: 0.66–0.86, *p* < 0.001), with an optimal cut-off value of 120, yielding a sensitivity of 72.4% and specificity of 70.3%. CACS predicted RV-FAC < 35% with an AUC of 0.73 (95% CI: 0.62–0.83, *p* < 0.001), and S′ < 10 cm/s with an AUC of 0.70 (95% CI: 0.59–0.81, *p* = 0.002). No statistically significant differences were observed between AUC values based on DeLong test comparisons (all *p* > 0.05) (Table 6, Figure 4).

**Figure 5:**
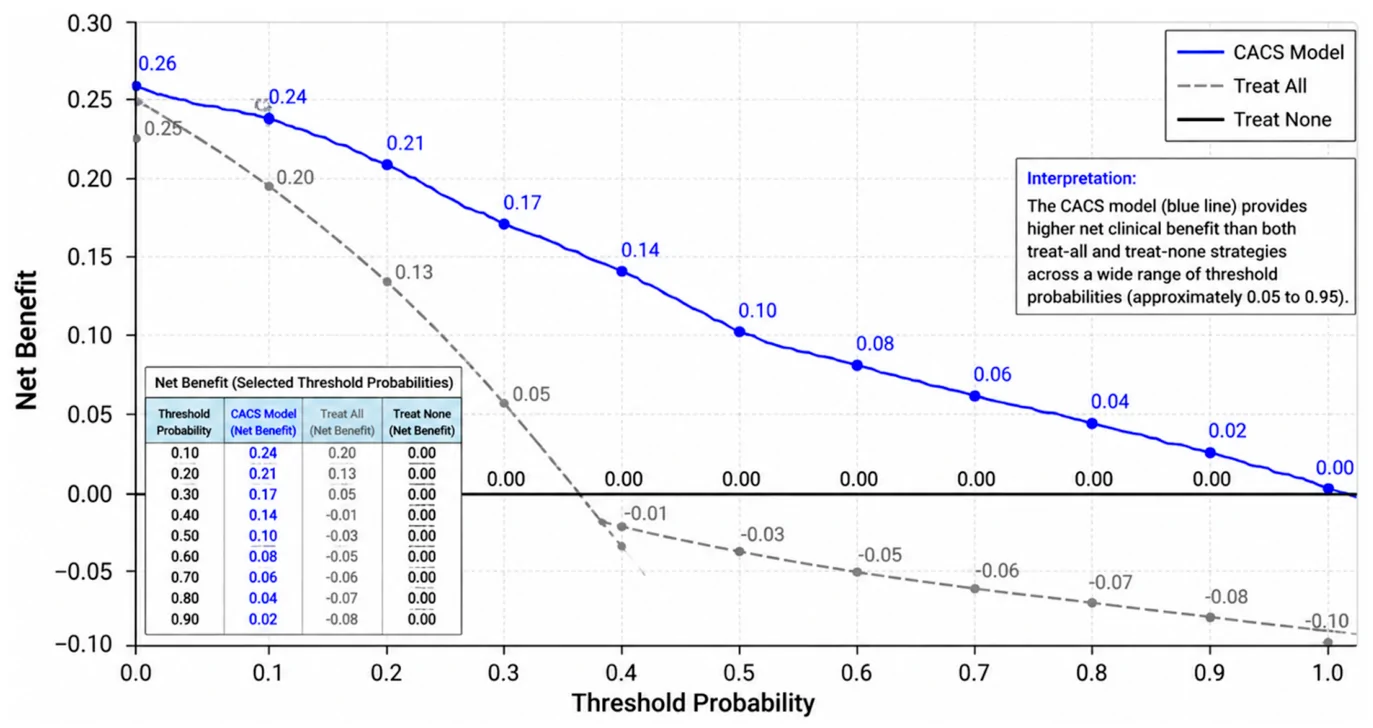
Decision curve analysis (DCA) for the potential clinical value of coronary artery calcium score (CACS) in predicting right ventricular dysfunction.

Exploratory DCA suggested a higher net benefit compared to both treat-all and treat-none strategies across a wide range of threshold probabilities. At a threshold probability of 0.10, the net benefit of the CACS model was 0.24 compared to 0.20 for the treat-all strategy. At thresholds of 0.20 and 0.30, the net benefit values for the CACS model were 0.21 and 0.17, respectively, whereas the corresponding values for the treat-all strategy were 0.13 and 0.05. As the threshold probability increased, the net benefit of the CACS model gradually decreased but remained consistently higher than that of the treat-all approach. At threshold probabilities of 0.50 and 0.70, the net benefit values for the CACS model were 0.10 and 0.06, respectively, while the treat-all strategy showed negative net benefit values. The treat-none strategy maintained a net benefit of zero across all threshold probabilities. At higher thresholds, including 0.80 and 0.90, the net benefit of the CACS model was 0.04 and 0.02, respectively, and approached zero at a threshold probability of 1.00 (Figure 5).

**Figure 6:**
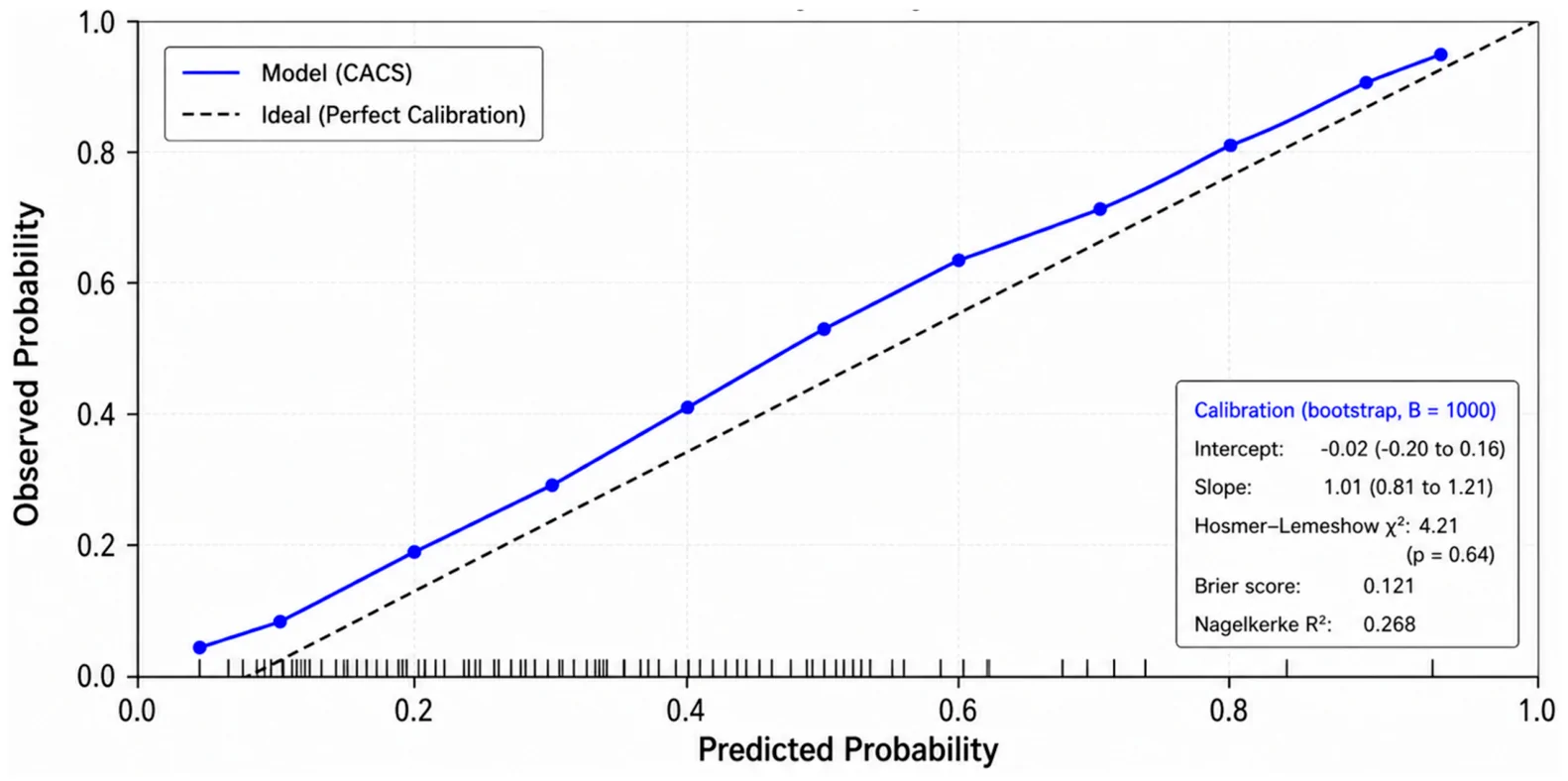
Bootstrap-calibrated plot of the model for predicting right ventricular dysfunction. Internal validation was performed using 1000 bootstrap resamples. The dashed line represents perfect calibration, while the solid line represents the model’s bootstrap-corrected calibration performance.

Internal validation using 1000 bootstrap resamples demonstrated acceptable calibration within the study cohort. The calibration intercept was −0.02 (95% CI: −0.20 to 0.16), and the calibration slope was 1.01 (95% CI: 0.81 to 1.21). The Hosmer–Lemeshow goodness-of-fit test was non-significant (χ^2^ = 4.21, *p* = 0.64), and the Brier score was 0.121 (Figure 6).

Subgroup analyses were performed according to hypertension status, diabetes mellitus, and age groups. The inverse relationship between CACS and RV function parameters was consistently observed across all subgroups. In patients with hypertension, CACS showed a significant negative correlation with TAPSE (r = −0.44, *p* < 0.001), RV-FAC (r = −0.38, *p* = 0.003), and S′ (r = −0.33, *p* = 0.008). Similarly, in patients without hypertension, negative correlations were observed between CACS and TAPSE (r = −0.39, *p* = 0.002), RV-FAC (r = −0.34, *p* = 0.006), and S′ (r = −0.29, *p* = 0.015). In the diabetes mellitus subgroup, CACS was negatively correlated with TAPSE (r = −0.41, *p* = 0.004), RV-FAC (r = −0.35, *p* = 0.009), and S′ (r = −0.30, *p* = 0.021), while in non-diabetic patients, similar associations were observed for TAPSE (r = −0.43, *p* < 0.001), RV-FAC (r = −0.37, *p* = 0.003), and S′ (r = −0.31, *p* = 0.010). Age-stratified analysis demonstrated that in patients aged <60 years, CACS was significantly correlated with TAPSE (r = −0.40, *p* = 0.001), RV-FAC (r = −0.35, *p* = 0.005), and S′ (r = −0.28, *p* = 0.018). In patients aged ≥60 years, stronger negative correlations were observed between CACS and TAPSE (r = −0.46, *p* < 0.001), RV-FAC (r = −0.39, *p* = 0.002), and S′ (r = −0.33, *p* = 0.007) (Table 7).

Reproducibility analysis demonstrated excellent agreement for both CACS and echocardiographic measurements. Interobserver ICC values were 0.95 (95% CI: 0.91–0.98) for CACS, 0.93 (95% CI: 0.88–0.97) for TAPSE, 0.91 (95% CI: 0.85–0.96) for RV-FAC, and 0.89 (95% CI: 0.82–0.95) for S′. Intraobserver ICC values were 0.97 (95% CI: 0.94–0.99), 0.95 (95% CI: 0.90–0.98), 0.94 (95% CI: 0.88–0.97), and 0.92 (95% CI: 0.86–0.96), respectively, indicating good-to-excellent reproducibility of all measurements.

Exploratory analyses of segmental coronary artery calcium scores demonstrated significant inverse correlations between LAD calcium score and TAPSE (r = −0.39, *p* < 0.001), RV-FAC (r = −0.34, *p* = 0.001), and S′ (r = −0.28, *p* = 0.008). Similarly, RCA calcium score was inversely correlated with TAPSE (r = −0.36, *p* < 0.001), RV-FAC (r = −0.31, *p* = 0.003), and S′ (r = −0.27, *p* = 0.010). LCx calcium score showed weaker correlations with TAPSE (r = −0.27, *p* = 0.009) and RV-FAC (r = −0.23, *p* = 0.025), whereas its correlation with S′ was not statistically significant (r = −0.19, *p* = 0.061). No significant correlations were observed between LM calcium score and TAPSE, RV-FAC, or S′ (all *p* > 0.05) (Table 8).

Exploratory multivariable analyses demonstrated that higher LAD calcium scores remained independently associated with lower TAPSE (β = −0.31, *p* < 0.001), RV-FAC (β = −0.28, *p* = 0.001), and S′ (β = −0.24, *p* = 0.004). Similarly, RCA calcium scores were independently associated with reduced TAPSE (β = −0.29, *p* < 0.001), RV-FAC (β = −0.25, *p* = 0.002), and S′ (β = −0.22, *p* = 0.007). In contrast, LM calcium score was not significantly associated with any RV functional parameter (all *p* > 0.05), whereas LCx calcium score showed only a modest association with TAPSE (β = −0.18, *p* = 0.029) but not with RV-FAC or S′ (both *p* > 0.05) (Table 9).

## 4. Discussion

In the present study, we demonstrated a significant and consistent association between CACS and RV systolic function parameters in patients with suspected stable coronary artery disease. Our findings indicate that increasing coronary atherosclerotic burden is associated with progressive impairment in RV function, as evidenced by reduced TAPSE, RV-FAC, and S′ values. Importantly, similar findings were observed across several complementary analytical approaches. However, these findings should be interpreted cautiously because of the relatively small sample size, particularly the limited number of patients in the high CACS group.

The relationship between coronary calcification and ventricular function has been widely investigated in the context of the left ventricle, but data regarding the right ventricle remain limited. Choi et al. reported that elevated CACS was significantly associated with the progression of left ventricular diastolic dysfunction, suggesting that coronary calcification reflects not only structural atherosclerotic burden but also functional myocardial impairment [25]. Studies by Gupta et al. and Cheong et al. emphasized that CACS is a comprehensive marker of subclinical cardiovascular damage beyond luminal stenosis [26,27]. Our findings extend these observations by demonstrating that coronary calcification is also associated with impairment in RV systolic function, supporting the concept of global cardiac involvement in atherosclerotic disease.

The clinical importance of RV dysfunction has been increasingly recognized in recent years. Haddad et al. highlighted that RV dysfunction is an independent determinant of morbidity and mortality across a broad spectrum of cardiovascular diseases [28]. Likewise, Sayour et al., in a meta-analysis, demonstrated that reduced TAPSE and RV-FAC are strongly associated with adverse cardiopulmonary outcomes [29]. Gentile et al. further confirmed the incremental prognostic value of RV functional parameters in chronic heart failure populations [30]. In agreement with these findings, our study showed that commonly used echocardiographic markers of RV systolic function progressively deteriorate with increasing CACS, suggesting that coronary atherosclerosis may influence RV performance even in the absence of overt clinical disease.

Several mechanisms have been proposed to explain the potential association between coronary atherosclerosis and RV dysfunction; however, these mechanisms were not directly investigated in the present study. Coronary artery disease may influence RV performance through impaired right coronary artery perfusion, whereas systemic atherosclerosis and microvascular dysfunction have also been suggested to contribute to myocardial remodeling and impaired ventricular function [31,32]. Accordingly, these mechanisms should be regarded as biologically plausible hypotheses rather than conclusions supported directly by our data. Severino et al. and Frąk et al. described how endothelial dysfunction and vascular inflammation contribute to myocardial remodeling and functional impairment [21,32]. In addition, Rahimi et al. demonstrated that arterial stiffness indices are independently associated with RV dysfunction in patients with coronary artery disease [31]. These findings support the hypothesis that RV dysfunction may represent a manifestation of systemic cardiovascular pathology rather than isolated right heart disease.

Another potential mechanism involves interventricular dependence. As demonstrated by Surkova et al., alterations in left ventricular function and filling pressures can significantly influence RV performance due to shared myocardial fibers and septal interactions [13]. Dini et al. also emphasized that increased left-sided pressures may lead to secondary RV dysfunction through pulmonary vascular changes [33]. Therefore, the observed association may be compatible with several previously proposed mechanisms, including ventricular interdependence and systemic cardiovascular remodeling. However, the present study was not designed to evaluate these mechanisms directly.

Additional exploratory analyses evaluating segmental coronary artery calcium burden demonstrated that LAD and RCA calcium scores showed stronger associations with RV systolic function than LM or LCx calcium scores. Both correlation and multivariable analyses consistently demonstrated inverse associations between LAD/RCA calcium burden and TAPSE, RV-FAC, and S′. Although these findings may reflect differences in the anatomical distribution of coronary calcification, coronary stenosis severity, lesion-specific ischemia, pulmonary artery pressure, and left ventricular function were not systematically evaluated. Accordingly, the territory-specific findings should be considered exploratory and hypothesis-generating and should not be interpreted as evidence of territory-specific coronary stenosis, ischemia, or a direct causal effect on RV function.

Our subgroup analyses further strengthen the validity of these findings by demonstrating consistent associations across clinically relevant patient groups. The persistence of significant correlations in patients with and without hypertension and diabetes mellitus suggests that the relationship between CACS and RV dysfunction is not solely driven by traditional cardiovascular risk factors. Moreover, the stronger correlations observed in older patients are consistent with previous studies indicating that age-related vascular changes may exacerbate myocardial dysfunction [12,34].

Secondary exploratory ROC analyses demonstrated AUC values ranging from 0.70 to 0.76 across the predefined echocardiographic thresholds, indicating moderate discrimination within the present cohort. This level of discrimination is broadly consistent with previous studies evaluating imaging-based biomarkers for functional cardiac outcomes [35,36]. DeLong tests showed no statistically significant differences among the AUCs for TAPSE, RV-FAC, and S′; however, given the limited sample size, the absence of statistically significant differences should not be interpreted as evidence of equivalent discriminatory performance. Bootstrap analysis suggested acceptable apparent calibration within the same cohort, while decision curve analysis indicated a potential net benefit across selected threshold probabilities. Nevertheless, these analyses were exploratory, were conducted using the same relatively small dataset, and lacked external validation. Accordingly, the reported cut-off values, calibration estimates, and net benefit findings should not be used for clinical decision-making. Furthermore, adjustment for the available covariates does not establish CACS as an independent determinant of RV dysfunction, as the observed associations may partly reflect unmeasured left ventricular dysfunction, pulmonary hemodynamic abnormalities, coronary stenosis severity, or other factors affecting RV performance.

A major strength of our study is the incorporation of advanced model evaluation techniques beyond conventional discrimination metrics. DCA suggested a potential net benefit across a range of threshold probabilities. However, given the retrospective design and limited sample size, these analyses should be considered exploratory and require external validation before clinical application. Similar findings have been reported in recent cardiovascular prediction studies, where DCA has been shown to provide valuable insights into the real-world applicability of diagnostic models [5,9]. Furthermore, bootstrap calibration analysis demonstrated acceptable agreement between predicted and observed probabilities within the study cohort. However, these findings should be interpreted cautiously because of the relatively small sample size and the absence of external validation. The discrimination and calibration analyses should therefore be considered exploratory.

Our findings provide preliminary evidence of an association between coronary calcium burden and conventional echocardiographic measures of RV systolic function. However, because the cohort was retrospectively selected and lacked comprehensive information on left ventricular function, pulmonary artery pressure, coronary stenosis severity, and advanced RV functional indices, no independent predictive or clinical role for CACS can be inferred from the present data. These observations should therefore be regarded as hypothesis-generating and require confirmation in prospective studies using comprehensive multimodality cardiovascular assessment [37,38].

This study has several limitations. First, its retrospective single-center design may limit the generalizability of the findings and precludes causal inference. In addition, the relatively small sample size, particularly the limited number of patients in the high CACS subgroup, may have reduced the statistical stability of subgroup analyses and predictive model performance estimates, thereby limiting the precision and clinical interpretability of subgroup-specific findings. Second, although all patients were analyzed as a single cohort using continuous CACS values as the primary analytical approach, comparisons across predefined CACS categories were exploratory and should be interpreted cautiously because baseline demographic and clinical characteristics may differ between these groups. Third, CCTA and transthoracic echocardiography were performed as part of routine clinical care rather than according to a standardized prospective research protocol, although only patients who underwent both examinations within a three-month interval were included to minimize temporal variability. Most importantly, left ventricular systolic and diastolic function parameters were not systematically available and therefore could not be incorporated into the multivariable models. Other potentially relevant variables, including pulmonary artery systolic pressure, coronary stenosis severity, and additional imaging-derived parameters, were likewise not consistently available. Given the physiological interdependence between the left and right ventricles, the present study cannot determine whether the observed association between CACS and RV systolic function is independent of concomitant left ventricular dysfunction. These omissions represent major potential sources of residual confounding. In addition, conventional echocardiographic parameters were used to assess RV systolic function. Advanced echocardiographic techniques, including right ventricular longitudinal strain, RV free-wall longitudinal strain, three-dimensional RV volumetric assessment, and right ventricular–pulmonary artery coupling indices (e.g., TAPSE/PASP), were not routinely available in this retrospective cohort and therefore could not be evaluated. These parameters may provide a more comprehensive assessment of right ventricular mechanics and RV–pulmonary vascular interaction and should be incorporated into future prospective studies. Finally, although additional exploratory analyses of segmental coronary artery calcium scores were performed, coronary stenosis severity, lesion-specific ischemia, and detailed quantitative plaque characterization were not systematically evaluated. Therefore, the observed segment-specific associations should be interpreted cautiously. External validation of the predictive models was not performed. Consequently, larger prospective multicenter studies, particularly those including a greater number of patients with high CACS values, are warranted to confirm these findings and establish their clinical applicability.

## 5. Conclusions

In this retrospective single-center cohort of selected patients with suspected stable coronary artery disease, higher CACS values were associated with lower conventional echocardiographic indices of right ventricular systolic function. Given the relatively small sample size, patient selection criteria, limited clinical and instrumental characterization, potential residual confounding, and absence of external validation, these findings are exploratory and do not establish CACS as a predictor of right ventricular dysfunction. Larger prospective multicenter studies incorporating comprehensive coronary, biventricular, and pulmonary hemodynamic assessments are required to confirm this association and determine whether it has any clinical relevance.

## Figures and Tables

**Table 1:** Demographic and clinical characteristics stratified by sex (*n* = 96).

Variable	Male (*n* = 62)	Female (*n* = 34)	Total (*n* = 96)	*p*-Value
Age (years)	57.8 ± 10.4	59.5 ± 11.2	58.4 ± 10.7	0.458
Body mass index (kg/m^2^)	27.5 ± 4.0	28.3 ± 4.5	27.8 ± 4.2	0.372
Hypertension, *n* (%)	32 (51.6)	22 (64.7)	54 (56.3)	0.216
Diabetes mellitus, *n* (%)	17 (27.4)	12 (35.3)	29 (30.2)	0.422
Dyslipidemia, *n* (%)	28 (45.2)	19 (55.9)	47 (49.0)	0.315
Chronic kidney disease, *n* (%)	7 (11.3)	5 (14.7)	12 (12.5)	0.749
COPD, *n* (%)	8 (12.9)	2 (5.9)	10 (10.4)	0.486
**Smoking status, *n* (%)**				
Current smoker	23 (37.1)	5 (14.7)	28 (29.2)	
Former smoker	17 (27.4)	5 (14.7)	22 (22.9)	
Never smoker	22 (35.5)	24 (70.6)	46 (47.9)	
**Medication use, *n* (%)**				
Beta-blockers	31 (50.0)	20 (58.8)	51 (53.1)	0.407
ACE inhibitors/ARBs	29 (46.8)	20 (58.8)	49 (51.0)	0.259
Statins	27 (43.5)	18 (52.9)	45 (46.9)	0.378
Antiplatelet agents	35 (56.5)	17 (50.0)	52 (54.2)	0.544
Calcium channel blockers	11 (17.7)	10 (29.4)	21 (21.9)	0.186

Continuous variables are presented as mean ± standard deviation, and categorical variables as *n* (%). Percentages in the male and female columns were calculated within each sex. Between-sex comparisons were performed using the independent-samples *t*-test for continuous variables and Pearson’s chi-square test for categorical variables. Fisher’s exact test was used for chronic kidney disease and COPD because of small expected cell counts. Smoking status is presented descriptively as current smoker, former smoker, and never smoker; no overall *p*-value is reported for this variable. COPD, chronic obstructive pulmonary disease; ACE, angiotensin-converting enzyme; ARB, angiotensin receptor blocker.

**Table 2:** Coronary CT characteristics and distribution of coronary artery calcium (*n* = 96).

Parameter	*n* (%) or Value
**Total CACS**	
Total CACS, median (IQR)	42 (5–185)
Total CACS, range	0–850
**CACS categories**	
Low, 0–99	64 (66.7)
No detectable calcium, CACS = 0	18 (18.8)
Minimal calcification, CACS = 1–10	22 (22.9)
Mild calcification, CACS = 11–99	24 (25.0)
Moderate, CACS = 100–399	20 (20.8)
High, CACS ≥ 400	12 (12.5)
**Coronary territory involvement**	
Left main involvement	15 (15.6)
Left anterior descending involvement	70 (72.9)
Left circumflex involvement	33 (34.4)
Right coronary artery involvement	43 (44.8)
**Segmental CACS among involved territories, median (IQR)**	
Left main	18 (8–38)
Left anterior descending	60 (18–185)
Left circumflex	35 (12–110)
Right coronary artery	45 (14–150)
**Number of territories with detectable calcification**	
No territory involved	18 (18.8)
One territory involved	28 (29.2)
Two territories involved	25 (26.0)
Three territories involved	17 (17.7)
Four territories involved	8 (8.3)

Values are presented as *n* (%) or median (interquartile range), as appropriate. A coronary territory was considered involved when its corresponding segmental Agatston score was >0. Segmental CACS values are presented only among patients with detectable calcification in the corresponding territory. The no-territory group corresponds to patients with a total CACS of 0. CACS, coronary artery calcium score; IQR, interquartile range.

**Table 3:** Echocardiographic parameters of right ventricular function (*n* = 96).

Parameter	Value (Mean ± SD)
TAPSE (mm)	18.4 ± 2.1
RV-FAC (%)	38.7 ± 6.2
S′ (cm/s)	16.0 ± 6.0
RV basal diameter (mm)	34.9 ± 3.8
RA major axis (mm)	44.2 ± 3.1
RA minor axis (mm)	32.8 ± 3.5
RA area (cm^2^)	20.4 ± 2.3

**Table 4:** Comparison of right ventricular function parameters across CACS categories (*n* = 96).

Parameter	Low CACS (0–99) (*n* = 64)	Moderate CACS (100–399) (*n* = 20)	High CACS (≥400) (*n* = 12)	*p*-Value
TAPSE (mm)	19.1 ± 1.8	17.8 ± 1.9	16.5 ± 2.0	0.001
RV-FAC (%)	41.2 ± 5.4	37.6 ± 5.9	34.1 ± 6.2	0.003
S′ (cm/s)	18.0 ± 5.0	15.0 ± 4.0	13.0 ± 4.0	0.012
RV basal diameter (mm)	33.8 ± 3.2	35.4 ± 3.6	37.1 ± 4.0	0.021
RA area (cm^2^)	19.6 ± 2.1	20.8 ± 2.4	22.1 ± 2.6	0.018

Values are expressed as mean ± standard deviation. Comparisons were performed using one-way ANOVA or Kruskal–Wallis test as appropriate.

**Table 5:** Multivariable associations of coronary artery calcium score with right ventricular systolic function parameters (*n* = 96).

**Dependent variable: TAPSE (mm)**						
**Variable**	**B**	**SE**	**β**	**95% CI for B**	***p*** **-value**	**VIF**
CACS	−0.004	0.001	−0.38	−0.006 to −0.002	<0.001	1.31
Age (years)	−0.042	0.017	−0.21	−0.075 to −0.009	0.015	1.24
Hypertension	−0.61	0.27	−0.17	−1.12 to −0.09	0.028	1.19
Diabetes mellitus	−0.55	0.26	−0.14	−1.05 to −0.05	0.041	1.17
**Model statistics:** Adjusted R^2^ = **0.34**, Model *p*-value **< 0.001**						
**Dependent variable: RV-FAC (%)**						
**Variable**	**B**	**SE**	**β**	**95% CI for B**	***p*** **-value**	**VIF**
CACS	−0.006	0.002	−0.33	−0.009 to −0.003	0.002	1.27
Age (years)	−0.065	0.028	−0.18	−0.110 to −0.020	0.021	1.21
Hypertension	−1.18	0.54	−0.16	−2.10 to −0.25	0.031	1.16
Diabetes mellitus	−1.06	0.52	−0.13	−1.95 to −0.18	0.044	1.14
**Model statistics:** Adjusted R^2^ = **0.29**, Model *p*-value **< 0.001**						
**Dependent variable: S′ (cm/s)**						
**Variable**	**B**	**SE**	**β**	**95% CI for B**	***p*** **-value**	**VIF**
CACS	−0.003	0.001	−0.29	−0.004 to −0.001	0.006	1.28
Age (years)	−0.017	0.008	−0.16	−0.030 to −0.005	0.033	1.22
Hypertension	−0.23	0.12	−0.12	−0.420 to −0.040	0.049	1.18
Diabetes mellitus	−0.20	0.11	−0.11	−0.390 to −0.030	0.052	1.16
**Model statistics:** Adjusted R^2^ = **0.24,** Model *p*-value **< 0.001**						

B, unstandardized regression coefficient; SE, standard error; β: standardized regression coefficient; CI: confidence interval; VIF: variance inflation factor. VIF values < 5 indicate no significant multicollinearity. Regression coefficients represent associations adjusted for the covariates shown in the table and should not be interpreted as establishing CACS as an independent predictor of RV dysfunction.

**Table 6:** ROC analysis of CACS for predicting right ventricular dysfunction.

Outcome (RV Dysfunction Definition)	AUC (95% CI)	Optimal CACS Cut-Off	Sensitivity (%)	Specificity (%)	*p*-Value
TAPSE < 17 mm	0.76 (0.66–0.86)	120	72.4	70.3	<0.001
RV-FAC < 35%	0.73 (0.62–0.83)	135	68.1	69.5	<0.001
S′ < 10 cm/s	0.70 (0.59–0.81)	140	65.2	67.8	0.002

AUC: area under the curve; CI: confidence interval. Optimal cut-off values were determined using the Youden index.

**Table 7:** Subgroup analysis of the correlation between CACS and right ventricular function parameters.

Subgroup	TAPSE (r, *p*-Value)	RV-FAC (r, *p*-Value)	S′ (r, *p*-Value)
Hypertension (+)	−0.44, <0.001	−0.38, 0.003	−0.33, 0.008
Hypertension (−)	−0.39, 0.002	−0.34, 0.006	−0.29, 0.015
Diabetes (+)	−0.41, 0.004	−0.35, 0.009	−0.30, 0.021
Diabetes (−)	−0.43, <0.001	−0.37, 0.003	−0.31, 0.010
Age < 60 years	−0.40, 0.001	−0.35, 0.005	−0.28, 0.018
Age ≥ 60 years	−0.46, <0.001	−0.39, 0.002	−0.33, 0.007

Values are presented as correlation coefficients (r) and *p*-values.

**Table 8:** Correlations between segmental coronary artery calcium scores and right ventricular function parameters.

Segmental CACS	TAPSE r (*p*)	RV-FAC r (*p*)	S′ r (*p*)
Left Main (LM)	−0.18 (0.081)	−0.15 (0.142)	−0.12 (0.236)
Left Anterior Descending (LAD)	−0.39 (<0.001)	−0.34 (0.001)	−0.28 (0.008)
Circumflex (Cx)	−0.27 (0.009)	−0.23 (0.025)	−0.19 (0.061)
Right Coronary Artery (RCA)	−0.36 (<0.001)	−0.31 (0.003)	−0.27 (0.010)

CACS, coronary artery calcium score; TAPSE, tricuspid annular plane systolic excursion; RV-FAC, right ventricular fractional area change; S′, tissue Doppler-derived systolic myocardial velocity.

**Table 9:** Association of segmental CACS with RV systolic dysfunction.

Segmental CACS	TAPSE β (95% CI)	*p*	RV-FAC β (95% CI)	*p*	S′ β (95% CI)	*p*
Left main (LM)	−0.08 (−0.19 to 0.03)	0.151	−0.07 (−0.21 to 0.06)	0.284	−0.05 (−0.16 to 0.05)	0.341
Left anterior descending (LAD)	−0.31 (−0.46 to −0.15)	<0.001	−0.28 (−0.43 to −0.12)	0.001	−0.24 (−0.39 to −0.08)	0.004
Left circumflex (LCx)	−0.18 (−0.34 to −0.02)	0.029	−0.15 (−0.31 to 0.01)	0.064	−0.12 (−0.27 to 0.03)	0.118
Right coronary artery (RCA)	−0.29 (−0.44 to −0.13)	<0.001	−0.25 (−0.40 to −0.09)	0.002	−0.22 (−0.37 to −0.06)	0.007

Standardized regression coefficients (β) are presented with 95% confidence intervals. Separate multivariable linear regression models were constructed for each right ventricular systolic function parameter. Models were adjusted for age, hypertension, and diabetes mellitus. CACS, coronary artery calcium score; CI, confidence interval; RV-FAC, right ventricular fractional area change; S′, tissue Doppler-derived systolic velocity; TAPSE, tricuspid annular plane systolic excursion.

## Data Availability

The original contributions presented in this study are included in the article. Further inquiries can be directed to the corresponding author.
